# Survival rates and reasons for revision of different stem designs in total hip arthroplasty for developmental dysplasia: a regional registry study

**DOI:** 10.1186/s10195-021-00590-y

**Published:** 2021-07-18

**Authors:** Alberto Di Martino, Francesco Castagnini, Niccolò Stefanini, Barbara Bordini, Giuseppe Geraci, Federico Pilla, Francesco Traina, Cesare Faldini

**Affiliations:** 1 Clinica Ortopedica E Traumatologica I, IRCCS Istituto Ortopedico Rizzoli, Via Pupilli, 1, 40136 Bologna, Italy; 2 Dipartimento di Scienze Biomediche e Neuromotorie - DIBINEM, University of Bologna, Bologna, Italy; 3 Ortopedia-Traumatologia E Chirurgia Protesica E Dei Reimpianti Di Anca E Ginocchio, IRCCS Istituto Ortopedico Rizzoli, Via Pupilli, 1, 40136 Bologna, Italy; 4 Laboratorio Di Tecnologia Medica, IRCCS Istituto Ortopedico Rizzoli, Via di Barbiano, 1/10, 40136 Bologna, Italy

**Keywords:** Tapered, Anatomic, Modular, Conical, Dislocation, Loosening, DDH, Dysplasia, Stem

## Abstract

**Introduction:**

Total hip arthroplasty (THA) in dysplastic hips is challenging, and each specific implant used in this context has been associated with specific complications. A registry study was performed to query survival rates, hazard ratios, and reasons for revision of different stem designs in THAs after developmental dysplasia of the hip.

**Materials and methods:**

A regional arthroplasty registry was inquired about cementless THAs performed for hip dysplasia from 2000 to 2017. Patients were stratified according to stem design in tapered (TAP; wedge and rectangular), anatomic (ANAT), and conical (CON), and divided on the basis of modularity (modular, M; nonmodular, NM). In total, 2039 TAP stems (548 M and 1491 NM), 1435 ANAT (1072 M and 363 NM), and 2287 CON (1020 M and 1267 NM) implants were included. Survival rates and reasons for revisions were compared.

**Results:**

The groups were homogeneous for demographics, but not fully comparable in terms of implant features. NM-CON stems showed the highest risk of failure (significant) and a high risk for cup aseptic loosening (2.5%). The adjusted risk ratio showed that NM-CON was more prone to failure (HR versus NM-ANAT: 3.30; 95%CI 1.64–7.87; *p* = 0.0003). Revision rates for dislocations and stem aseptic loosening did not differ between cohorts.

**Conclusions:**

NM-CON stems showed the highest risk of failure, especially high rates of cup aseptic loosening. NM-CON implants were not more prone to dislocations and stem aseptic loosening. Clinical comparative studies are required to investigate the causes of NM-CON failures, which may be due to abnormal acetabular morphology or imperfect restoration of the proximal biomechanics.

## Introduction

Total hip arthroplasty (THA) after developmental hip dysplasia (DDH) is a challenging procedure owing to bone and soft-tissue abnormalities [1]. Despite the notable anatomical variations, THAs after DDH did not show inferior outcomes compared with THAs in primary osteoarthritis in long-term follow-up studies [2–4].

Engesaeter et al. highlighted that the choice of implant significantly affected the risk of revision in THAs after DDH [2]. The choice of the stem in THAs after hip dysplasia is still controversial. Many different stem designs have been proposed, with different fixation levels (from the more proximal-engaging short stems to the distal anchoring conical implants) and with different settings of femoral antetorsion (from the more bounded metaphyseal filling stems to the free adjustment of the conical implants) [5]. Stem choice in hip dysplasia is based on the need to bypass the proximal–distal mismatch, to perform in some cases a femoral shortening osteotomy, to control the combined anteversion, and to fit into small-sized femurs [5, 6]. No less important is the restoration of the proximal femoral biomechanics, carried out by the combining cup and femoral stem positioning, to reconstitute a physiological geometry [5, 6].

Clinical and radiological outcomes of THA surgery in DDH patients are extremely heterogeneous. While most of the case series involving just one prosthetic model report good or excellent results, only few case–control studies are available [7–9]. Moreover, it is difficult to determine the best implant with respect to the dysplastic morphology in large populations. The only three comparative studies retrieved in literature provide few inconclusive findings: better osseointegration was achieved with extensively coated stems, and the control of the anteversion with tapered wedge implants was more problematic and less predictable compared with metaphyseal filling stems [7–9].

Therefore, a registry study involving cementless THAs after DDH was designed. Stems were stratified according to the main features (design, modularity) as provided by manufacturers. The aims were to (1) compare the survival rates, (2) evaluate the hazard ratios (HRs) for failure, and (3) assess the reasons for revision and compare the survival rates using stem-focused endpoints (revisions for stem aseptic loosening and revisions for implant dislocations).

## Materials and methods

The Register of the Orthopaedic Prosthetic Implants (Registro dell’Implantologia Protesica Ortopedica, RIPO) is an Italian regional (Emilia-Romagna, 4.5 million inhabitants) arthroplasty registry following hip, knee, and shoulder procedures since 2000 [10]. Paper data forms about demographic features, diagnoses, and types (batch and code) of the implants in primary and revision surgeries are regularly filled out by the surgeons and actively collected by the RIPO registry [10]. To avoid possible loss to follow-up, analysis has been limited to Emilia-Romagna resident patients, because even if revision surgery would be performed outside the region, the cost of the procedure would be billed back to Emilia-Romagna and therefore trackable and accounted as a failure. Capture rate of the registry is 98% (missing data accounting for 2%). The accuracy is confirmed by crossover comparisons and missing data retrievals [10]. RIPO structure is similar to the main national arthroplasty registries [10]. When an implant failed, the surgeon performing the revision surgery fills a specific RIPO form, specifying the reason for revision, the batch and the code of the newly implanted device, and the demographical data of the patients receiving the reimplantations.

The RIPO database was inquired about cementless THAs performed for developmental dysplasia of the hip between January 2000 and December 2017. The diagnosis of DDH was provided by the surgeon filling in the RIPO form: no dislocation degree (i.e., Crowe grade) was provided. The exclusion criteria were: metal-on-metal implants with heads ≥ 36 mm, resurfacing hips, and patients not residing in the Region (to maximize adhesion to follow-ups, as specified in previous papers) [10].

The stems of the included THAs were stratified according to implant design and modularity. These data were provided by the single manufacturers.

To avoid excessive data fragmentation with obvious consequences on the statistical analysis and the reliability of the report, three main implant designs were identified (according to Khanuja et al.): anatomic (6), tapered cone (3B), and tapered wedge/rectangular (2, 3A, and 3C) [11]. The distinction between the tapered cone stems and the tapered wedge/rectangular was made owing to the fixation (proximal diaphyseal for conical implants and metaphyseal and metaphyseal/diaphyseal for tapered devices) and the possibility to adjust the anteversion (complete for conical implants and limited for tapered devices) [5, 6, 11]. Extensively coated, cylindrical stems were not included owing to the poor number of implants in registry. To avoid small, scarcely informative cohorts, a binomial classification of modularity (modular and nonmodular) was adopted, without including the site and the type of modular junctions.

**Fig. 1:**
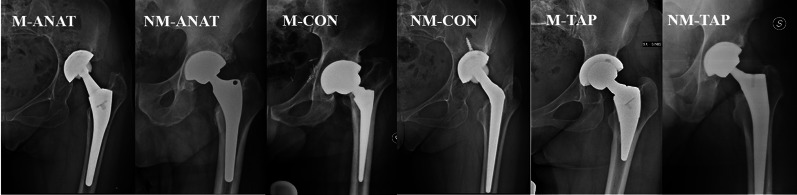
A representative stem model was provided for every cohort. M-ANAT: Ancafit, Cremascoli Ortho; NM-ANAT: AptaFix, Adler Ortho; M-CON: Acuta, Adler Ortho; NM-CON: Wagner Cone, Zimmer; M-TAP: SMF, Smith & Nephew; NM-TAP: Corae, Adler Ortho

Six cohorts were identified (Fig. 1): modular anatomic (M-ANAT), nonmodular anatomic (NM-ANAT), modular tapered cone (M-CON), nonmodular tapered cone (NM-CON), modular tapered wedge/rectangular (M-TAP), and nonmodular tapered wedge/rectangular (NM-TAP). Demographics and implant-related features of the six cohorts were collected and compared (Table 1).

**Table 1:** Demographics were homogeneous in the six cohorts, whereas implant-related features were not similar

Demographics and implant features						
	M-ANAT	NM-ANAT	M-CON	NM-CON	M-TAP	NM-TAP
Number of implants	1072	363	1020	1267	548	1491
Mean age (years)	58.3	59.8	58.3	58.9	61	63
Female gender (%)	79	70.5	82.9	80.4	75.4	71
BMI (kg/m^2^) between 19 and 25 (%)	48.8	39.3	48.5	46.5	45.7	39.9
BMI (kg/m^2^) > 30 (%)	11.9	14.7	12.8	13.8	12.3	18.1
Most used implants						
Stems	AncaFit (528) Apta (503) Sam-Fit (20)	ABG II (151) CFP (48) Fit (37)	Modulus (578) Alata Acuta (364) Profemur C (51)	Conus (932) ADR (70)	Recta (170) Hydra (111) EHS (45)	SL-Plus (159) Taperloc (110) CLS (98)
Cups	AncaFit (501) Fixa Ti-Por (330) Fixa (169)	ABG II (142) Delta PF (31) Top (29)	Fixa Ti-Por (273) Delta TT (272) Delta PF (254)	Protek SC (146) Continuum (96) CLS (95)	Fixa Ti-Por (187) Fixa (115) AncaFit (100)	EP-Fit Plus S and N (180) R3 (90) EP-Fit Plus Endoplus (85)
Cup type						
Cementless, press-fit	44.1%	64.5%	65.1%	56.7%	59.5%	63.9%
Cementless, press-fit, with wings	38.8%	1.7%	3.4%	13.0%	15.5%	6.1%
Cementless, press-fit, with screws	17.1%	33.1%	31.5%	23.4%	25.0%	25.9%
Cementless, external thread	0.0%	0.8%	0.0%	6.8%	0.0%	4.1%
Bearing surfaces						
CoC	80.7	53	85.1	22.0	67	45.9
CoP	15.2	17.1	11.1	32.9	23.2	27.5
MoP	3.9	24	3.6	22	7.8	15.4
MoM	0	0.8	0	20.6	0.4	4.2
Head size < 36 mm (%)	85.3	73.2	61.6	89	72.6	64.4
Follow-up (years)	10.7	9.4	6.5	10.1	8.5	6.7
Implants at risk at 10 years (%)	59.9	53.4	23.2	53.7	37.4	28.5

The first aim of the study was the assessment of the survival rates at long-term follow-ups, the endpoint being revision for any cause. The second aim was the evaluation of the hazard ratio (HR) for implant failure, adjusted for age and gender. The third aim was the assessment of the reasons for revision of the six cohorts by comparison of the survival rates of the six cohorts, with stem-calibrated endpoints: revisions for stem aseptic loosening and revisions for dislocations. Accordingly, HRs adjusted for age and sex were compared.

Institutional board review was waived as data collection is a regional standard practice and the identity of the patients is concealed.

### Statistical analysis

Statistical analyses were performed using the software JMP, version *12.0.1*. SAS Institute Inc., Cary, NC, 1989–2007. A descriptive analysis about patient demographics, implant features, and reasons for revision was conducted providing means, ranges, and percentages. *t*-Test or *χ*^2^ test was used to compare the values. Kaplan–Meier survival analysis was performed; each curve was flanked by another two curves, the 95% confidence interval. The endpoints were: the revision of any single component, stem aseptic loosening, dislocations, and cup aseptic loosening. The endpoint was specified for each curve. Survival times of unrevised implants were calculated considering the last date of observation (31 December 2017) or the date of death. The survival rates of the six cohorts were compared using the Wilcoxon test. HR was tested using the Schoenfeld residual method; age at surgery and sex used for adjustment fulfilled the proportional hazard assumption for the whole period. The threshold for significance was *p* = 0.05 for all the tests.

## Results

### Study cohorts

In total, 5761 THAs were considered eligible for the study; of them, 45.8% (2640) implants were modular. After stem stratification for design and modularity, six cohorts were obtained, of which NM-TAP was the largest one (Table 1). Four groups out of six involved more than 1000 implants. The M-ANAT encompassed 1072 THAs, all with a modular neck–stem junction. The NM-ANAT accounted for 363 cases. The M-CON cohort included 1020 THAs, while the NM-CON group had 1267 THAs. The M-TAP cohort included 548 THAs, and NM-TAP involved 1491 implants.

The main demographic and implant-related features are provided in Table 1. The six groups were homogeneous for demographics [age, gender, and body mass index (BMI)], whereas the distribution of implant-related features, i.e., bearing surfaces and head sizes, was not similar (*p* > 0.05). M-CON showed the highest rate of ceramic-on-ceramic couplings (85.1%), whereas NM-ANAT had the highest proportion of metal-on-polyethylene bearings (24%). Considering all the involved THAs, the vast majority of couplings were ceramic-on-ceramic (56.5%), followed by ceramic-on-polyethylene (22.4%). The proportion of head size ≥ 36 mm was the highest in the M-CON (38.4%), followed by NM-TAP (35.6%) and M-TAP (27.4%). The lowest rate of heads ≥ 36 mm was in the NM-CON (11%). The total amount of heads ≥ 36 mm was 25.4%.

### Survival rates

**Fig. 2:**
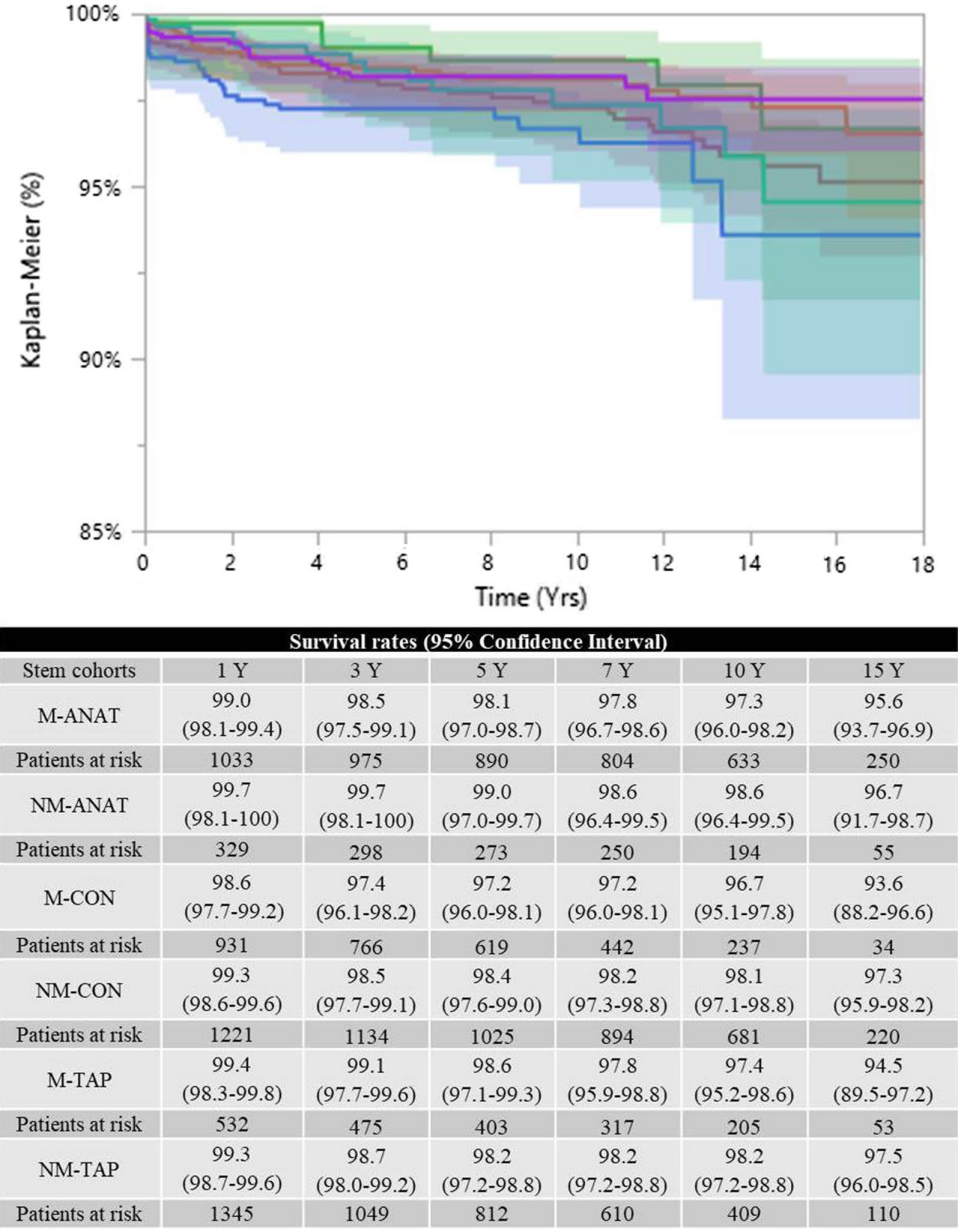
NM-CON achieved the lowest survival rates (*p* < 0.05, Wilcoxon test; endpoint: any revision). M-ANAT: red line; NM-ANAT: green line; M-CON: blue line; NM-CON: orange line; M-TAP: cyan line; NM-TAP: violet line

The survival rates (using any revision surgery as an endpoint) showed that the NM-ANAT group achieved the best results, whereas the NM-CON cohort achieved the worst outcomes at long-term follow-up (*p* < 0.05, Wilcoxon test) (Fig. 2). Specifically, the NM-ANAT cohort achieved a survival rate of 98.4% (95% CI 96.1–99.3), whereas 94.4% of the NM-CON stems survived at 10 years (95% CI 92.9–95.7).

### Hazard ratios (HRs) for failure

When the model was adjusted for age at surgery and gender, the NM-CON stems were more at risk of failure than NM-ANAT (HR 3.30, 95% CI 1.64–7.87, *p *= 0.0003) and M-ANAT (HR 1.93, 95% CI 1.33–2.84, *p* = 0.0004). The M-CON stems were more prone to failure than NM-ANAT (HR 2.79, 95% CI 1.32–6.85, *p* = 0.0056) and M-ANAT (HR 1.63, 95% CI 1.03–2.56, *p* = 0.0340). NM-TAP cohort was more predisposed to failure than M-ANAT (HR 1.67, 95% CI 1.12–2.52, *p* = 0.0112) and NM-ANAT (HR 2.87, 95% CI 1.40–6.90, *p* = 0.0025).

### Reasons for revision

In Table 2 is reported the distribution of the causes for revision surgery. M-ANAT stems mainly failed because of periprosthetic fractures (0.6%), the NM-ANAT because of global aseptic loosening, periprosthetic infections, and dislocations (all 0.6%), and the M-CON because of stem aseptic loosening (0.9%). Cup aseptic loosening was the most frequent reason for revision in the NM-CON (2.5%), accounting for more than one-third of all the failures in the group. M-TAP stems tended to fail because of stem aseptic loosening, implant breakage, and periprosthetic fractures (0.5%). The most notable reasons for revision in the NM-TAP cohort were cup aseptic loosening and dislocations (0.9%).

**Table 2:** Reasons for revisions are listed as percentages of the total cohort and as distribution of the failures: Cup aseptic loosening in NM-CON showed a very high incidence

	M-ANAT		NM-ANAT		M-CON		NM-CON		M-TAP		NM-TAP	
Reasons for revision	Percentage (%)	Distribution of the failures (%)	Percentage (%)	Distribution of the failures (%)	Percentage (%)	Distribution of the failures (%)	Percentage (%)	Distribution of the failures (%)	Percentage (%)	Distribution of the failures (%)	Percentage (%)	Distribution of the failures (%)
Stem aseptic loosening	0.3	8.6	0	0	0.9	29	0.5	22.2	0.5	21.4	0.1	8.3
Recurrent dislocations	0.4	11.4	0.3	16.7	0.6	19.4	0.2	7.4	0.2	7.1	0.4	25
Global aseptic loosening	0.2	5.7	0.6	33.3	0.1	3.2	0.2	7.4	0.4	14.3	0.2	12.5
Periprosthetic infection	0.2	5.7	0.6	33.3	0.1	3.2	0.2	7.4	0	0	0.1	4.2
Periprosthetic fracture	0.5	14.3	0.3	16.7	0.4	12.9	0.3	14.8	0.5	21.4	0.3	16.7
Prosthetic breakage	0.5	14.3	0	0	0.2	6.5	0	0	0.5	21.4	0.1	4.2
Pain without loosening	0.1	2.9	0	0	0.1	3.2	0	0	0	0	0.1	8.3
Primary instability	0.1	2.9	0	0	0.2	6.5	0	0	0	0	0	0
Polyethylene wear	0.1	2.9	0	0	0	0	0	0	0	0	0	0
Other	1	21.3	0	0	0.5	16.1	0.9	40.8	0.4	14.3	0.2	12.5
Total	3.3	100	1.7	100	3	100	2.1	100	2.6	100	1.6	100

### Survival rates by stem-focused endpoints

**Fig. 3:**
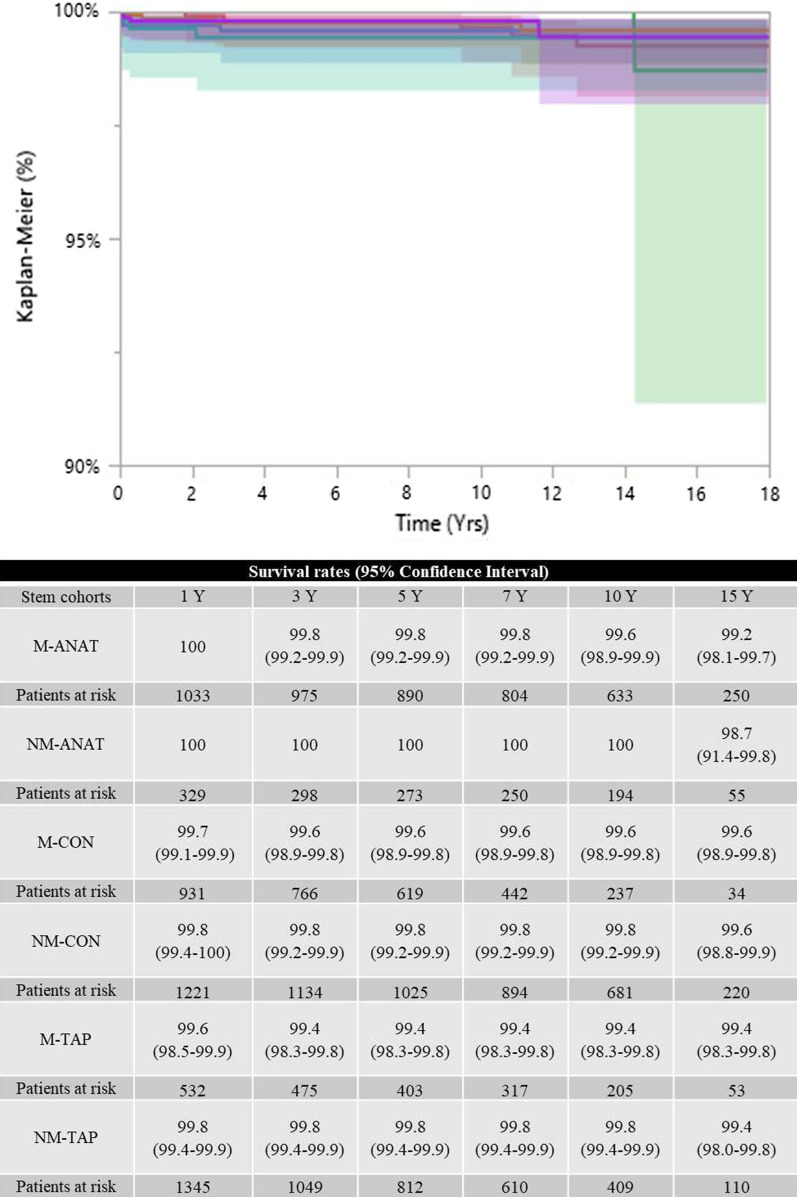
The six survival rates were similar when the endpoint was revision due to stem aseptic loosening (*p* = 0.0462, close to significance). M-ANAT: red line; NM-ANAT: green line; M-CON: blue line; NM-CON: orange line; M-TAP: cyan line; NM-TAP: violet line

**Fig. 4:**
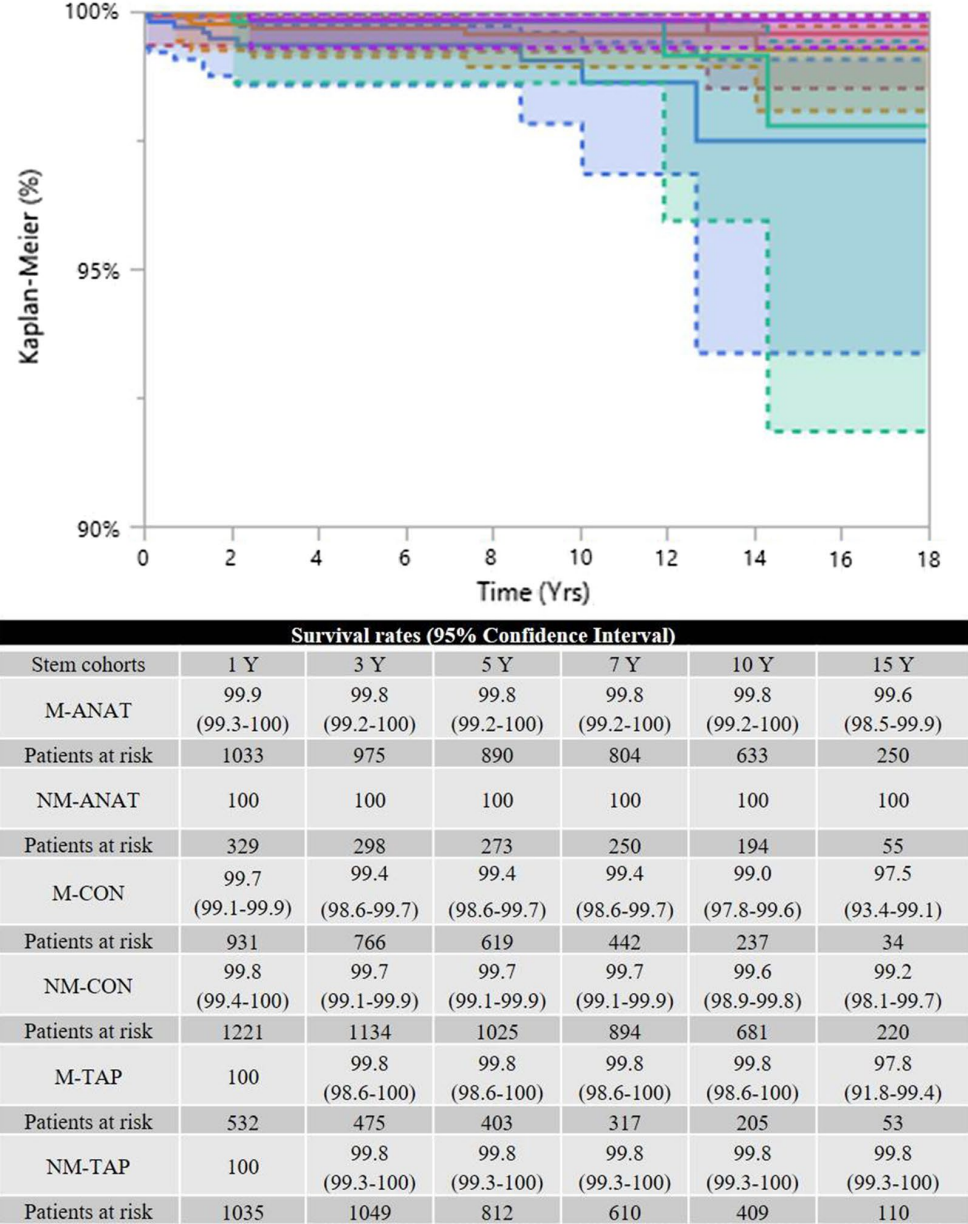
The six survival rates were similar when the endpoint was revision due to dislocations (*p* = 0.2571). M-ANAT: red line; NM-ANAT: green line; M-CON: blue line; NM-CON: orange line; M-TAP: cyan line; NM-TAP: violet line

When revisions due to stem aseptic loosening was the endpoint, the six cohorts achieved similar outcomes (borderline result, *p *= 0.0462, Wilcoxon test): M-CON and NM-CON were the worst-performing groups (Fig. 3). When the model was adjusted for age at surgery and gender, no significant differences were recorded (*p* > 0.05). When the endpoint was revision surgery for dislocation, the six cohorts were comparable (*p* = 0.2571, Wilcoxon test; Fig. 4).

## Discussion

NM-CON stems in cementless THAs after dysplasia showed lower survival rates and higher adjusted risks of failure. The rate of cup aseptic loosening was high in comparison with all the other cohorts and accounted for more than one-third of all the revisions of NM-CON implants. However, when stem-focused endpoints were adopted (revisions for stem aseptic loosening and for dislocations), NM-CON achieved dependable performances, not inferior to the other groups.

The main limitations of this report are related to the nature of the registry study. It is not possible to assess the preoperative conditions and the postoperative outcomes from clinical and radiological perspectives, including Crowe or Hartofilakidis classification. This is a relevant flaw for the stem cohorts, as no stratification according to the preoperative classification of dysplasia was conducted. This should be considered approaching the results obtained by the current research, and surely other studies based on clinical severity of the DDH are needed to enlarge the knowledge on stem survivorship. Moreover, conservatively treated complications of the implants (with special regard to dislocations) could not be captured. However, it is the first paper providing a full, comprehensive comparison of different cementless stems in THAs after hip dysplasia in a sizeable cohort of patients, at long-term follow-up. A few considerations about the design of the present study should be made. Stem classification was not as comprehensive and complete as the one proposed by Khanuja et al. in order to avoid small, uninformative groups [11]. Due to the features of the implants and the anatomical variations imposed by DDH, the use of fully comparable groups for implant-related features (bearings and head diameter) would have resulted in an unreliable statistical analysis. Despite the different follow-ups of the cohorts, more than 20% of implants per group were still at risk at 10 years, making the curves fully reliable at long term (Table 1) [12].

Despite the limitations, the present report highlighted that all the groups, apart from the NM-CON stems (which missed the benchmark by a few decimals, 94.4%), achieved a survival rate higher than 95% at 10 years, as required by the 2014 NICE guidelines for end-stage hip osteoarthritis [13]. These data confirm the dependable outcomes of every cohort of stems and, as a collateral finding, of THA procedures in DDH, confirming the findings of other registry reports [2–4]. Differently from older similar studies, the present report described much higher survival rates, probably reflecting the evolution of surgical skills and implant technology [2, 3].

The results provided by this study confirm the good long-term outcomes achieved with anatomic and tapered stems in DDH, in agreement with previous reports [14–16]. As NM-ANAT and NM-TAP stems may only partially address high femoral antetorsion, small-sized femurs, and proximal–distal mismatches, these implants should be limited to modest dysplastic anatomies [14, 16]. The modular options may provide some benefits, but some device-specific complications may be encountered [14, 17].

NM-CON cohort achieved a significantly lower performance at long term; this finding was evident when both the crude survival rates and the sex- and age-adjusted HRs were assessed. Adjusted HRs specifically highlighted that the risk for NM-CON stems to fail was two-to-threefold higher than that for ANAT implants. Interestingly, a very large proportion (more than one-third) of failures in NM-CON was due to cup aseptic loosening (2.5%), differently from the results from other cohorts (TAB survival). Such incidence may have notably influenced the survival rates and even the adjusted HRs. In fact, when calibrated endpoints as revisions for stem aseptic loosening and dislocations were considered, NM-CON showed dependable performances at long term, with no differences with the other cohorts. Two hypotheses can be made: the first is related to the preoperative anatomic variations due to DDH. As NM-CON stems allow a free setting of the femoral anteversion, and the highest subluxations according to Crowe classification tend to display a higher femoral anteversion, these implants may have been preferred in these complex cases with severe morphological abnormalities of the femur and acetabulum [18, 19]. Thus, the preoperative dysplastic anatomy may have exerted a notable impact on the survival rates of the cohort, more on the acetabular side than on the femoral side, causing a high rate of socket failures [20]. However, the lack of radiographic assessment prevents us from being definitive about this supposition.

The latter hypothesis concerns the biomechanical hip reconstruction provided by NM-CON stems. NM-CON implants may have achieved lower performances in nonfocused survival rates owing to the inferior restoration of the hip biomechanics with respect to other stems, exerting influences more on socket fixation than on dislocation rates [20]. However, since radiographic data were not available from the registry, the issue is unanswered. It is likely that both the situations may have concurred with the high rate of cup aseptic loosening in absence of any significant cause of failure more closely related to the stem itself.

In conclusion, this registry study enlightened that THA in DDH maintain excellent results of survivorship, independently by the choice of the stem. NM-CON stems resulted as possible low-performing implants at long term. However, stem-calibrated endpoints (loosening and dislocations) did not confirm this finding. Moreover, the high incidence of cup aseptic loosening in NM-CON suggests that the native acetabular and femoral morphologies, or the inadequate reconstruction of proximal hip biomechanics, may have played a role. Conclusions are limited by the intrinsic nature of the registry study, which does not allow us to correlate the patient with the severity of the dysplasia: for this reason, case–control studies with radiographic assessments are required to confirm these findings and draw a definitive conclusion.

## Data Availability

Data are collected from RIPO and are available online (http://ripo.cineca.it/authzssl/index.htm) or at Rizzoli Orthopaedic Institute Medical Technology Laboratory.

## Abbreviations

THA: Total hip arthroplasty
DDH: Developmental hip dysplasia
RIPO: Register of the Orthopaedic Prosthetic Implants
M-ANAT: Modular anatomic
NM-ANAT: Nonmodular anatomic
M-CON: Modular tapered cone
NM-CON: Nonmodular tapered cone
M-TAP: Modular tapered wedge/rectangular
NM-TAP: Nonmodular tapered wedge/rectangular
HR: Hazard ratio
BMI: Body mass index
